# Do We Really Need Additional Contrast-Enhanced Abdominal Computed Tomography for Differential Diagnosis in Triage of Middle-Aged Subjects With Suspected Biliary Pain

**DOI:** 10.1097/MD.0000000000000546

**Published:** 2015-02-20

**Authors:** In Kyeom Hwang, Yoon Suk Lee, Jaihwan Kim, Yoon Jin Lee, Ji Hoon Park, Jin-Hyeok Hwang

**Affiliations:** From the Department of Internal Medicine, Seoul National University College of Medicine, Seoul National University Bundang Hospital, Seongnam-si (IKH, YSL, JK, J-HH); Department of Internal Medicine, Wonkwang University College of Medicine, Wonkwang University Sanbon Hospital, Gunpo (IKH) and Department of Radiology, Seoul National University College of Medicine, Seoul National University Bundang Hospital, Seongnam-si, Republic of Korea (YJL, JHP).

## Abstract

Enhanced computed tomography (CT) is widely used for evaluating acute biliary pain in the emergency department (ED). However, concern about radiation exposure from CT has also increased. We investigated the usefulness of pre-contrast CT for differential diagnosis in middle-aged subjects with suspected biliary pain.

A total of 183 subjects, who visited the ED for suspected biliary pain from January 2011 to December 2012, were included. Retrospectively, pre-contrast phase and multiphase CT findings were reviewed and the detection rate of findings suggesting disease requiring significant treatment by noncontrast CT (NCCT) was compared with cases detected by multiphase CT.

Approximately 70% of total subjects had a significant condition, including 1 case of gallbladder cancer and 126 (68.8%) cases requiring intervention (122 biliary stone-related diseases, 3 liver abscesses, and 1 liver hemangioma). The rate of overlooking malignancy without contrast enhancement was calculated to be 0% to 1.5%. Biliary stones and liver space-occupying lesions were found equally on NCCT and multiphase CT. Calculated probable rates of overlooking acute cholecystitis and biliary obstruction were maximally 6.8% and 4.2% respectively. Incidental significant finding unrelated with pain consisted of 1 case of adrenal incidentaloma, which was also observed in NCCT.

NCCT might be sufficient to detect life-threatening or significant disease requiring early treatment in young adults with biliary pain.

## INTRODUCTION

The proverb “the abdomen is the physician's grave,” quoted in one study, vividly explains how challenging it is to make an accurate diagnosis in patients complaining of abdominal pain.^1^ In fact, abdominal pain is a common complaint, accounting for 6.5% of causes for visits to the emergency department (ED).^2^ Among myriad of differential diagnosis considered in such patients, stone-related biliary diseases manifest with a comparatively typical nature. Although clinical information with laboratory findings is helpful for evaluating biliary diseases, proper management based only on clinical and laboratory assessment does not seem sufficient. Therefore, imaging studies remain crucial for differential diagnosis.^3–4^ Since the early 1970s, the number of computed tomography (CT) examiantions has progressively increased in clinical practice, especially in the ED.^5–8^ Recent data showed CT examinations increased by 330% between 1996 and 2007 in the ED, especially in work-ups for abdominal pain by almost 1000% during the same period.^2^ Actually several reports showed data supporting the usefulness and effectiveness of enhanced CT in ED for diagnostic accuracy.^9–11^

However, as the use of CT increases dramatically, concern about radiation exposure from CT has grown and many reports on the radiation hazard from CT have also increased. One study reported an estimated potential life time risk of radiation-related cancer of 0.05%.^12^ Particularly, Smith-Bindman et al^13^ demonstrated that the median effective radiation dose for multiphase abdomen pelvic CT was the highest with 31 millisievert (mSv) and also revealed that the development of radiation-induced cancer per CT examination was the greatest in women at age 20 years with 1 in 250 Therefore, in the era of widespread and routine use of CT, though admitting the role of CT scan for accurate diagnosis in the ED, the diagnostic modality should be more carefully considered on the basis of benefits and potential risks. When managing patients with suspected biliary disease in the ED, we doubt whether multiphase CT is an appropriate answer for assessing biliary system in middle-aged patients who have a low chance of malignancy but greater chance of vulnerability to radiation. Therefore, we investigated whether non-contrast CT (NCCT) is comparable with multiphase CT in patients under age 50 with suspected biliary disease in the ED setting.

## PATIENT AND METHOD

### Design

**FIGURE 1:**
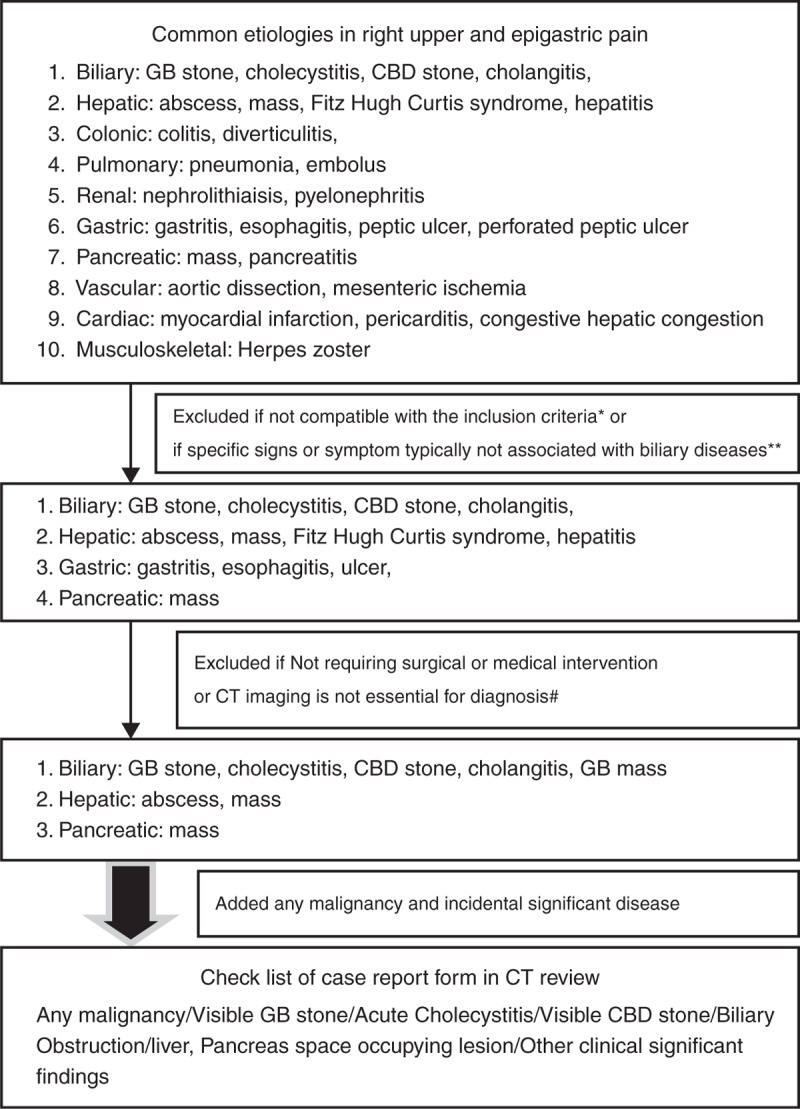
Flow diagram for selecting target disease and completing case report form. Only common conditions in young adults presenting with right upper quadrant or epigastric pain in the emergency department were selected. Diseases that were common in patients with older than 60 years or condition with distinct feature from biliary tract disease were excluded. Also only significant conditions requiring a surgical or medical intervention were included then the target diseases only when computed tomography (CT) is considered to be essential in making diagnosis and helpful for planning the management were selected. Finally, case report form for reviewing the CT was completed. ^∗^Diverticulitis, aortic dissection, mesenteric ischemia (common in elderly with mean age over 60), pancreatitis (elevation of amylase, lipase), myocardial infarction, hepatic congestion (elevation of cardiac enzyme), pulmonary embolism (d-dimer elevation), nephrolithiasis, pyelonephritis (hematuria, pyuria), perforated peptic ulcer, pneumonia (X-ray abnormality). ^∗∗^Herpes zoster (skin lesion), pericarditis (typical chest pain, dyspnea, EKG change). ^#^Esophagitis, gastritis, colitis, peptic ulcer, hepatitis.

Over 2 years from January 2011 to December 2012, all patients between 20 and 50 years of age who visited the ED for biliary pain and who underwent multiphase CT with a pancreatobiliary CT protocol (PBCT) were investigated. The study was approved by the Seoul national university bundang hospital institutional review board. We thoroughly reviewed all electronic medical records. Suspected biliary pain was typically defined as: severe, steady pain located in the epigastrium or the right upper quadrant lasting >30 min; findings of abnormal liver function test (LFT) (total bilirubin ≥2 mg/dL or alkaline phosphatase or γ-glutamyl transpeptidase or aspartate aminotransferase or alanine aminotransferase >1.5 standard deviations [STD]) or right upper quadrant (RUQ) tenderness/Murphy's sign on physical examination.^14,15^ We excluded patients older than 50 years or younger than 20 years, those with amylase or lipase levels >3 times the upper normal limits, those with a history of malignancy or significant cardiovascular diseases, and those with advanced liver diseases. We also excluded patients who visited the ED for trauma or who had been referred due to suspected malignancy. We selected target diseases for the study based on a flow diagram (Fig. 1). Briefly, target diseases were chosen based on textbooks or journals.^16–18^ Consequently, 7 diseases including gallbladder (GB) stones, acute cholecystits, common bile duct (CBD) stones, acute cholangitis, liver abscess, liver mass, and pancreas mass remained as target conditions for this study. Finally, we added any incidental malignancy or significant findings not associated with pain (Fig. 1). Final diagnoses were confirmed by pathologic reports undergoing surgery or by reviewing electronic medical records during follow-up. The clinical relevance of the final diagnosis was considered to be either *“*significant” or *“*non-significant” regardless of association with pain.^19^ “Significant” causes were defined as a condition requiring significant therapeutic management and subclassified as Group A: life-threatening malignancy necessitating therapeutic actions, Group B: significant diseases requiring surgical or medical intervention with indubitable clinical or prognostic relevance. “Non-significant” etiologies were defined as conditions requiring only conservative management and assigned to either group C: conditions requiring clinical awareness, follow-up however not necessitating intervention, Group D: findings not requiring follow-up or further tests. All NCCTs were firstly reviewed by 2 expert radiologists (YLand JHK) according to case report form. After a washout period of 8 weeks, all PBCTs, including NCCT, were reviewed again by same radiologists. Only the initial clinical information was provided before reviews were made. Rate of NCCT was evaluated for each of the 4 groups with reference to PBCT. The primary end point was the probable rate of overlooking group A by NCCT with reference to PBCT. The secondary end point was the rate of overlooking group B by NCCT with reference to PBCT.

### CT Image Acquisition

Patients underwent multiphase CT scans comprising noncontrast, pancreatic parenchymal, and portal venous phases using 64- (Briliance 64, Philips Healthcare) or 256-detector row (iCT256, Philips Healthcare) machines in the supine position and in a craniocaudal direction. All patients received nonionic iodinated contrast material (Iomeron 350; 2 mL/kg; Bracco Diagnostics Inc.) with a rate of 3 mL/s. An automatic bolus tracking technique was used to initiate the CT scans after contrast material injection. A region-of-interest circle was placed by the radiologist in the abdominal aorta at the origin of the celiac axis, and the triggering threshold was set at 200 Hounsfield units. The time delays for the initiation of pancreatic parenchymal phase and portal venous phase scans were 15 and 60 s, respectively. In all patients, the Z-axis scanning range of noncontrast and pancreatic parenchymal phase images covered from 4 cm above the liver dome to the inferior tip of segment 6 of the liver. For portal venous phase images, the Z-axis scanning range covered from 4 cm above the liver dome to the lower margin of the sacroiliac joint.

### Statistical Analysis

A 2-sided 95% confidential interval for difference in detection rate of life-threatening condition or significant disease between PBCT and NCCT was calculated for the probability of the overlooking rate. The Pearson *χ*^2^ test and Fisher exact test were used to determine the difference of etiologies for biliary pain between groups classified according to age, a history of biliary stone (including GB and CBD stone), clinical significance. All statistical analyses were performed using SPSS 21 version (IBM Corporation, Armonk, NY,). A 2-sided *P* value <0.05 was considered to be statistically significant.

## RESULTS

### Baseline Patient Characteristics

Of a total of 319 subjects, 183 patients met the inclusion criteria of the study. The baseline characteristics are shown in Table 1. There were 98 men and 90 women with a mean age of 38 years. Twenty-eight subjects were febrile and 15 patients had jaundice. Weight loss was identified in only 3 individuals. Mean duration from development of pain to visiting the ED was 3 days. Twenty-six percent of the patients (48/183) had medical history of gallstones. On physical examination, 80% (147/183) of patients revealed RUQ tenderness, whereas only 30% (56/183) showed Murphy sign. Other laboratory findings include liver function test and inflammatory markers are shown in Table 1.

**TABLE 1:**
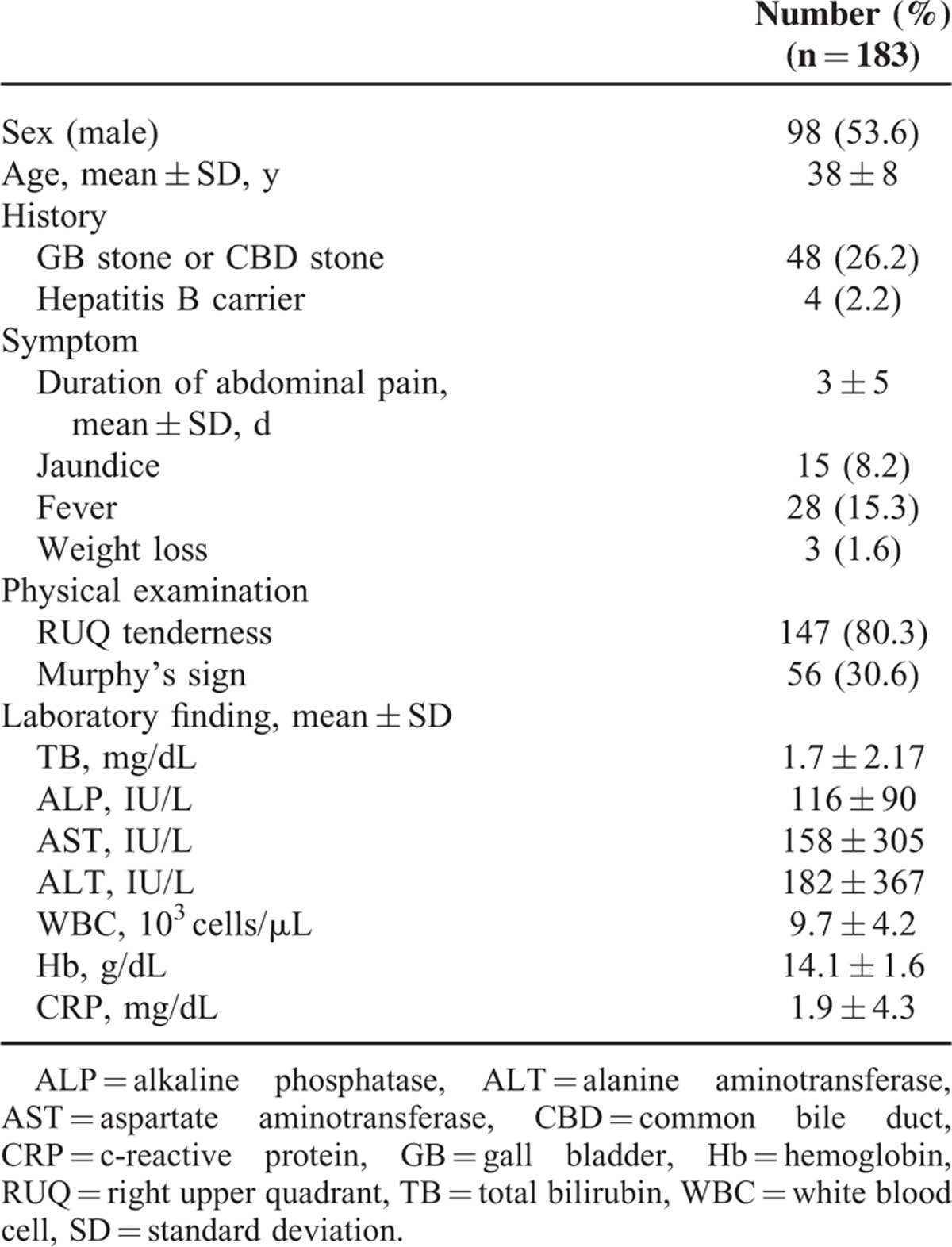
Baseline Characteristics of Total 183 Patients

### Final Diagnoses of 183 Patients With Suspected Biliary Pain

Etiology of suspected biliary pain is presented in Table 2. 66.7% of 183 patients were diagnosed finally as biliary diseases including stone-related pain, cholecystitis, or cholangitis. Nineteen patients (10.4%) had hepatic problems: 15 acute hepatitis, 3 liver abscess, and 1 large hemangioma requiring resection. Two patients had pain from gastric or intestinal origin (1 gastric ulcer and 1 acute appendicitis). One patient with Fitz-Hugh-Curtis syndrome complained of RUQ pain with elevated LFT without vaginal discharge. Two patients visited the ED for muscular tear. One had pain from gallbladder malignancy. The definite cause of pain was not identified in 36 patients (19.7%).

**TABLE 2:**
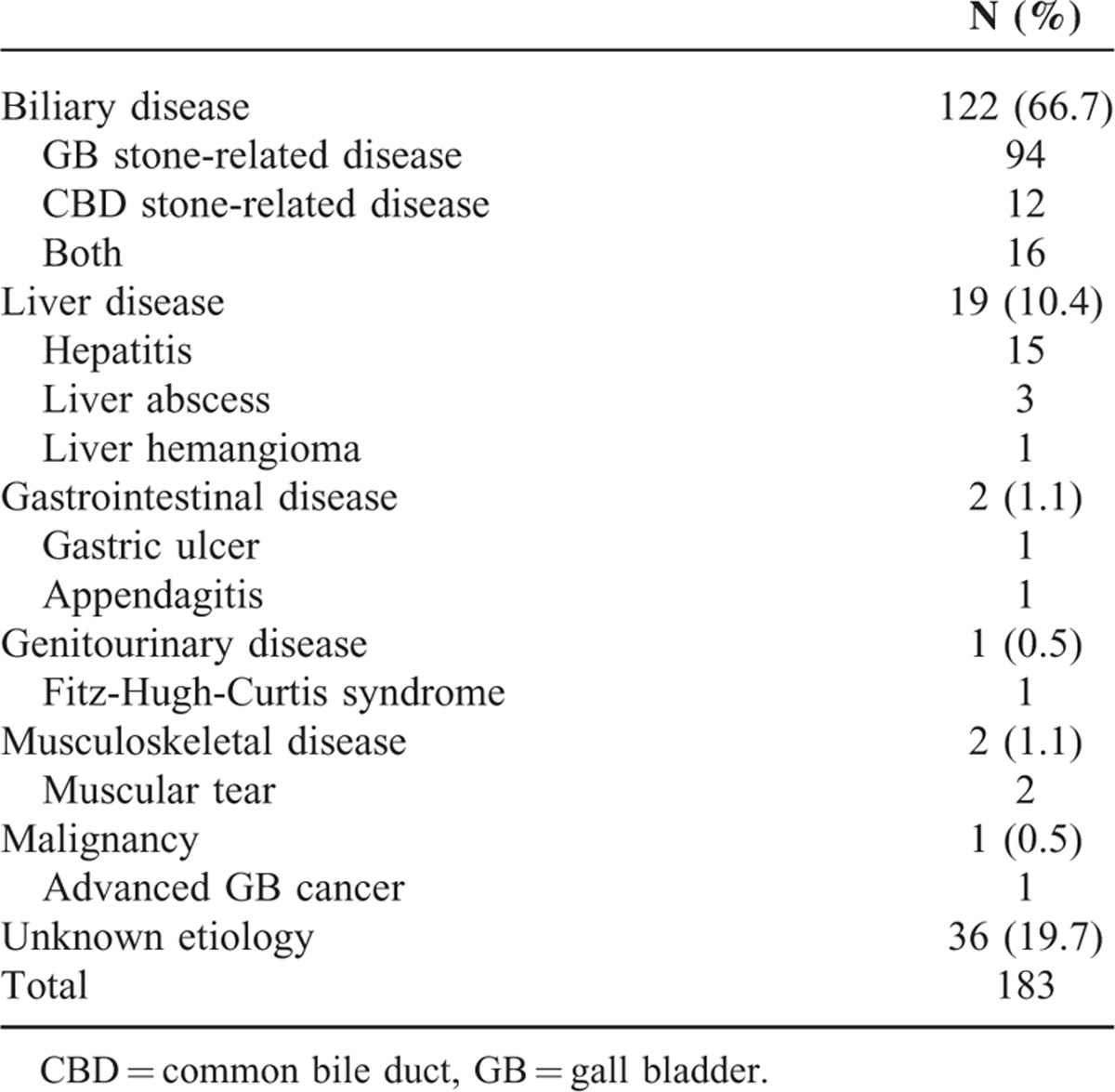
Etiology of Suspected Biliary Pain

### Etiologic Grouping According to Clinical Significance

**FIGURE 2:**
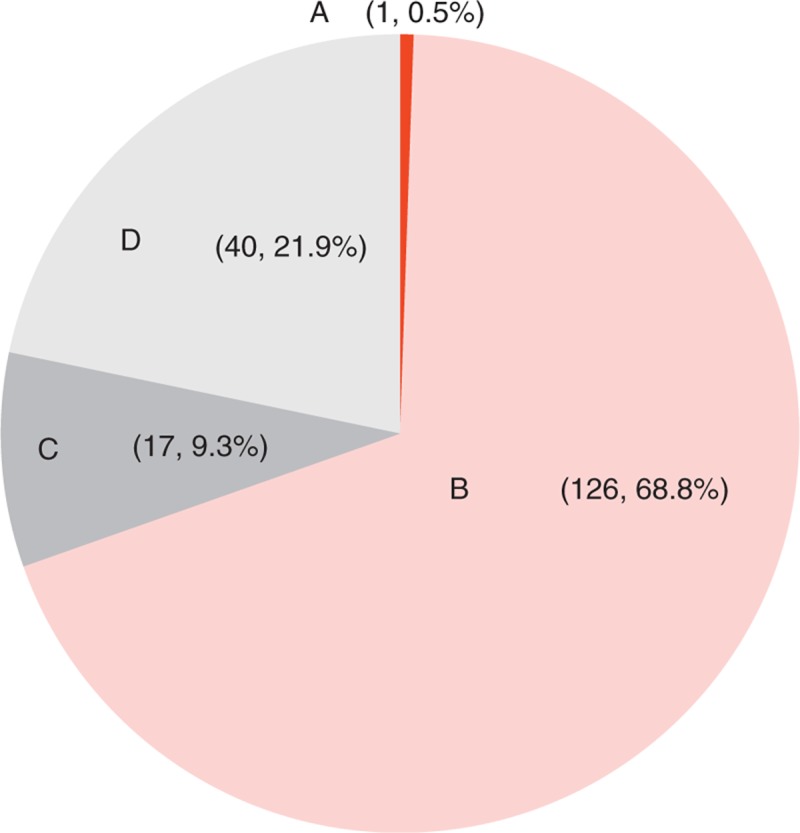
Etiology of suspected biliary pain according to clinical significance. The overall final etiologic groupings were classified into 4 categories: life-threatening conditions (group A), significant diseases requiring intervention (group B), significant diseases requiring follow-up (group C), and insignificant conditions (group D).

The overall final etiologic groupings according to clinical significance are described in Fig. 2. In the study, Group A contained only 1 case of gallbladder cancer (0.5%) out of a total of 183 subjects. Group B included GB stone, CBD stone, acute cholecystitis, acute cholangitis, liver abscess, a huge liver hemangioma, which caused RUQ pain (68.8%). Group C consisted of hepatitis, gastric ulcer, appendicitis, Fitz-Hugh-Curtis syndrome (9.3%). Group D included muscular tear or no definite etiology (21.9%). Approximately 70% of total subjects had a *“*significant” condition. When comparing clinical relevance, according to age and presence of history with biliary stone disease, patients aged between 40 and 49 years had more *“*significant” diseases than those 40 years or younger (81.9% vs 58.0%, *P* = 0.001) (unpublished data). In the subgroup with a history of biliary stone, 44 of 48 (91.7%) had a *“*significant” condition, as opposed to 82 of 135 (60.7%) subjects without a history of biliary stone (*P* < 0.001) (unpublished data).

### Comparison in NCCT and PBCT

As mentioned previously, 7 criteria for comparing CT finding between NCCT and PBCT were reviewed (Table 3). Among 183 subjects, 1 patient was diagnosed with advanced gallbladder cancer. The PBCT findings of the lesion was enhanced irregular GB mass with infiltration into the cystic duct and periportal lymph node, causing diffuse dilatation of common hepatic and intrahepatic duct dilatation. The findings were comparable in NCCT showing an irregular GB mass with diffuse biliary obstruction suggestive of GB malignancy. Considering there was only 1 case of malignancy out of a total of 183 subjects, the estimated probable overlooking of life-threatening disease without contrast enhancement was calculated to be 0% to 1.5%. Comparisons for other significant findings for diseases requiring treatment are also presented in Table 3. NCCT was effective on finding of GB stones, CBD stones, and liver Space occupying lesions (SOLs). The calculated probable overlooking rate was 10% for visible GB stone, 5.3% for visible CBD stone, and 3.0% for liver SOL. Findings of acute cholecystitis and biliary obstruction were more confirmatory by PBCT. Six equivocal cases of cholecystitis by NCCT were changed into 5 positive and 1 negative cholecystitis and 7 equivocal cases of biliary obstructions were interpreted as 5 positive biliary obstructions and 2 negative ones after contrast enhancement. The calculated probable overlooking rate for acute cholecystitis and biliary obstruction was 6.8% and 4.2%, respectively. Incidental findings, which were unrelated with biliary pain but required treatment, were seen in 1 case by PBCT. The lesion was an adrenal incidentaloma 1.1 cm in length that revealed an aldosterone-producing tumor on a functional study. The tumor was also observed in NCCT and stochastically the chance of missing a significant lesion is <1.5%. Other incidental finding was not significant conditions including fatty liver, liver cyst, or simple renal cyst.

**TABLE 3:**
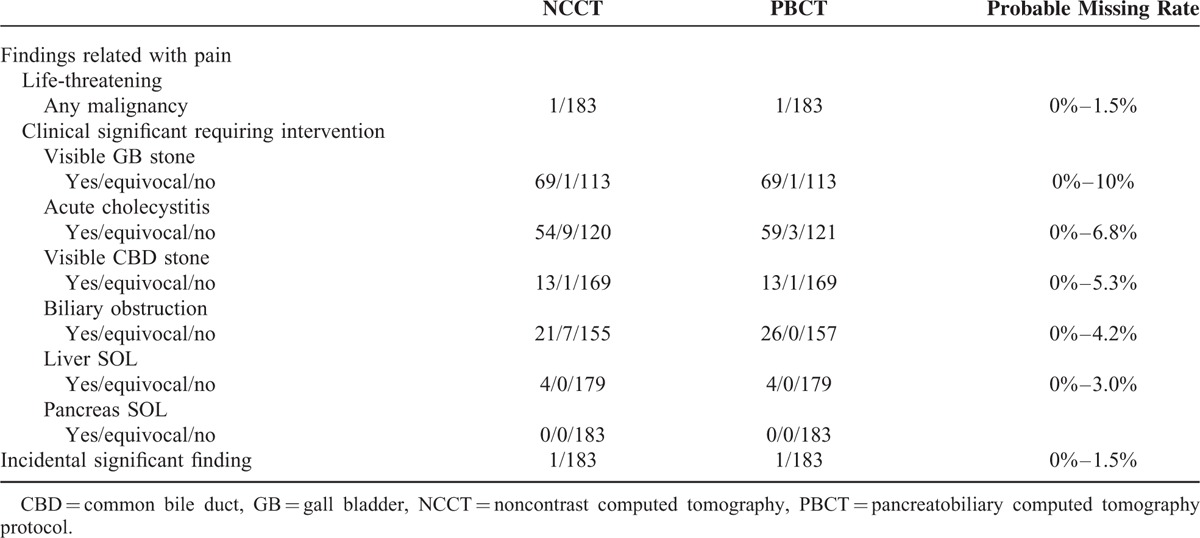
Comparison of Findings in NCCT and PBCT

## DISCUSSION

Biliary tract disease is one of the most common cause of acute abdominal pain presenting to the emergency department and abdominal ultrasound (US) has been recommended as the first test for evaluating biliary disease. However, US may occasionally be limited in the evaluation of distal common bile duct or whole pancreas due to overlying bowel gas or large body habitus. However, as CT has no limitations in visualization of the biliary tree and is able to even demonstrate other unexpected causes of abdominal pain, CT is now widely used for evaluating the patients with abdominal pain in everyday clinical practice.

The number of CT examinations has rapidly grown during the last decades and they involve almost all medical practices. In particular, one-third of CT scans are performed in the ED.^20–23^ There are multi-factorial reasons for making CT a common modality in the ED, including expanded indications of CT and high diagnostic value resulting from technologic advances, increased availability of CT scanners, and time constraints caused by limited bedside evaluation^5–24^ When managing a patient rushed to the ED for abdominal pain, generally physician thought of 2 important conditions to exclude. First, a diagnosis of surgical emergency, constituting urgent referral such as obstruction and peritonitis, should be considered. Even after ruling out surgical emergency, another concern, the probability of missing malignancy, remains. However, the prevalence of malignancy is known to be quite low in adults less than 50 in all organs, including the hepatobiliary system.^25–26^ Therefore, the benefits from multiphase CT with additional contrast and radiation exposure should be contemplated in relatively young people.

In the present study, we identified several results. First, it was verified that malignancy in the hepatobiliary and pancreatic system is rare in patients younger than <50 years even with suspected biliary pain. Only 1 life-threatening condition (advanced GB cancer) was diagnosed, on both PBCT and NCCT. The statistical probability of overlooking due to not using contrast enhancement was <1.5%. Although the arithmetical possibility for overlooking malignancy on NCCT may exist, clinical and laboratory information could be complementary for suspected malignancy in addition to imaging modality. Taking the very low malignancy rate under age 50 as well as the usefulness of clinical and laboratory information into consideration, the actual overlooking of life-threatening condition with NCCT is much lower than simple arithmetic calculations in the real clinical world. Second, it was found that NCCT was comparable with PBCT in the detection of significant diseases requiring intervention. All findings with biliary stones and liver SOLs were identified equally on NCCT. Six cholecystitis and 7 biliary obstructions were equivocal in NCCT compared with PBCT. Even though PBCT might clarify these findings, additional contrast use did not affect decision making for performing therapeutic intervention such as cholecystectomy or endoscopic retrograde cholangiography because GB or CBD stone was almost always accompanied with cholecystitis or cholangitis, giving an important clue for making the treatment plan. Also endoscopic ultrasound or magnetic retrograde pancreatocholangiogram might be useful in achieving more information without exposure to additional radiation. Third, our data showed that only 1 significant condition (adrenal aldosterone-producing tumor) was incidentally detected on both NCCT and PBCT. The probable overlooking rate was 1.5% at maximum. Based on the results from our study, NCCT seems sufficient for evaluation of biliary pain in patients under age 50 years.

According to the National Council on Radiation Protection and Measurement in 2009, radiation exposure from medical procedures increased 6 times from 0.53 mSv in 1987 to 3.00 mSv in 2006 in the United States and CT was one of the main factors for the increase.^21^ Our data showed that most young patients who were suspected to have biliary pain and had experienced biliary stones diseases eventually were diagnosed as biliary stone disease, which could be easily detected on NCCT. This means our cohort does not need to be exposed to additional radiation only for diagnosis in this setting. Growing utilization of CT did raise concerns about not only unnecessary radiation exposure but also contrast-related complications. Even there was no case in our study, recent reports showed the allergic reaction rate was from 0.2% to 0.6% and the severe complication rate was from 0.002% to 0.01% of a total of intravenous contrast injection.^27–29^ Although it is expected that there is a very low possibility of severe reaction, the event can be life-threatening and once it occurs, patients rapidly deteriorate and die.^28^ Therefore, our data may give an opportunity to reconsider whether multiphase CT by unconditional reflex could be justified, in case of young adults with typical biliary pain in ED setting. Among the several limitations in our study, it was a retrospective study with data collected from electronic medical records. Second, 2 radiologists reviewed images of NCCT and PBCT, and although there was an intervening wash-out period to minimize recall memory, the preceding review of NCCT might influence the subsequent findings of PBCT. Despite such limitations, the authors suggest that NCCT is comparable with PBCT to detect life-threatening disease or significant disease requiring early treatment in young adults with acute pain of suspected biliary origin. We investigated whether NCCT might not be inferior to PBCT in patients younger than 50 years with suspected biliary pain in the ED setting in the study. Our data showed that the risk of malignancy in the liver and pancreatobiliary system was negligible, as previously reported. Furthermore, the authors found that NCCT was comparable with PBCT to detect life-threatening disease or significant disease requiring early treatment in this clinical setting.
